# Virtual Residency Interviews: What Variables Can Applicants Control?

**DOI:** 10.7759/cureus.14938

**Published:** 2021-05-10

**Authors:** Stefano Tassinari, Laura C Perez, Anibal La Riva, Aref S Sayegh, Peter Ullrich, Chitang Joshi

**Affiliations:** 1 Department of Surgery, Division of Plastic & Reconstructive Surgery, Northwestern University Feinberg School of Medicine, Chicago, USA; 2 Urology, University of Southern California Institute of Urology & Catherine and Joseph Aresty Department of Urology, Keck School of Medicine, University of Southern California, Los Angeles, USA

**Keywords:** virtual interview, the match, residency, fellowship, covid-19

## Abstract

Due to the ongoing coronavirus disease 2019 (COVID-19) pandemic, almost all residency programs have adopted virtual interviewing for the National Residency Matching Program® (NRMP) or The Match® 2021. Hence, applicants have had to adapt quickly to this process, since the interviewers and the applicants were mostly inexperienced regarding this process. To date, program directors have had a successful experience on this new modality, and since the pandemic continues to limit in-person meetings and given the benefits that virtual interviews provide in terms of transportation, booking, and cost, there is a high chance that interviews for The Match 2022 will also be conducted in the same, virtual way. In light of this, we performed a review of the literature by using PubMed, Embase, Scopus, and other online resources to analyze certain critical aspects and offer recommendations for residency and fellowship applicants to improve their performance in virtual interviews. Despite the current surge of virtual interviewing in today’s technology-driven era, virtual interviewing programs for residency and fellowship candidates selection are still in their infancy. We have learned that applicants can control certain aspects such as technology, settings, dress code, and behavior so that they can tailor their experience to make it more favorable and fulfilling. Ensuring proper preparation in terms of the variables that can influence the virtual experience is key for a successful interview.

## Introduction and background

The coronavirus disease 2019 (COVID-19) pandemic has led to unprecedented global disruptions in the field of academic medicine. The National Residency Matching Program® (NRMP) or The Match® has not been immune to this, especially with regard to interviewing applicants for residency programs. Due to the ongoing pandemic, residency and fellowship programs had migrated from an in-person to a virtual platform to select candidates in order to avoid potential asymptomatic spread and minimize risk to individuals not required for patient care. This challenging scenario had demanded flexibility and brought about a shift in priorities as students faced unprecedented levels of uncertainty regarding curricular requirements, licensing exams, and the residency match process [1].

Virtual interviews have been used in the past by some programs for selecting residents and fellows. The Ophthalmology Department at the University of Arizona offered virtual interviews for their candidates during The Match® 2011. Almost half of the candidates (43%) chose to make use of this option through the Skype platform. Later, a study was conducted to determine if choosing virtual interviews over in-person ones influenced the ranking of the candidates, which revealed that there were no statistical differences between the two groups in terms of interview outcomes. Twelve candidates out of the 25 ranked positions were found to be chosen from the virtually interviewed group. Also, a faculty survey was carried out to determine the interviewees' experience and to find out if they would consider virtual interviews in the future. It revealed that all of them had a positive experience and responded positively to the prospect of attending virtual interviews in the future [2].

Furthermore, virtual interviews have several advantages/benefits compared to in-person interviews, such as minimizing applicants' stress levels and reduced travel expenses. Additionally, remote interviews are well comparable to in-person interviews in terms of expression of ideas and communication [3]. Therefore, authorities who conduct the training programs are confident in the virtual interview's feasibility and ability to evaluate the candidates' capability and determine their interpersonal skills. Consequently, it is now even more imperative from the applicants' standpoint to fine-tune preparations and manage variables that could enable them to optimize the virtual experience. In the present article, we present four key elements that may influence the outcome of a residency program virtual interview and provide some tips to the applicants to improve their overall experience based on a comprehensive review of the literature.

## Review

Strategies to improve interview performance

Vining et al. (2018) have put forward several recommendations based on their study of general surgical oncology fellowship virtual interviews [4]. One suggestion is to conduct a "mock interview day". While this is beneficial for many reasons, there is the added necessity in virtual interviews to identify connectivity and audio issues and anticipate technology-based problems that may occur at both the applicant's and faculty member's end during the actual interview. They have proposed an interview structure consisting of an introductory session, virtual rooms with residents and other applicants, one-on-one sessions with program directors and assistant professors, and concluding with a question-answer session, which is not mandatory.

Technology

Candidates should have access to and get familiarized with the pertinent software platform before the interview day. Usually, programs will disclose the details of the software to be used in correspondence with the candidate prior to the interview. Applicants should test for adequate internet bandwidth. At least 800 kbps/1.0 Mbps (up/down) is recommended for high-quality video [5]. However, bandwidth greater than 25 Mbps is optimal.

In order to avoid the battery being exhausted during videoconferencing, computers should be kept plugged into the power source throughout the interview. All unnecessary program windows and app notifications should be closed and turned off to avoid interruptions and distractions. However, if technical problems are encountered, notification to the protocol staff should be made immediately. In cases of network connection issues, applicants can stop and reconnect again. However, if difficulties persist throughout the entire interview, candidates can request another interview as per the protocol of each program [3].

**Figure 1:**
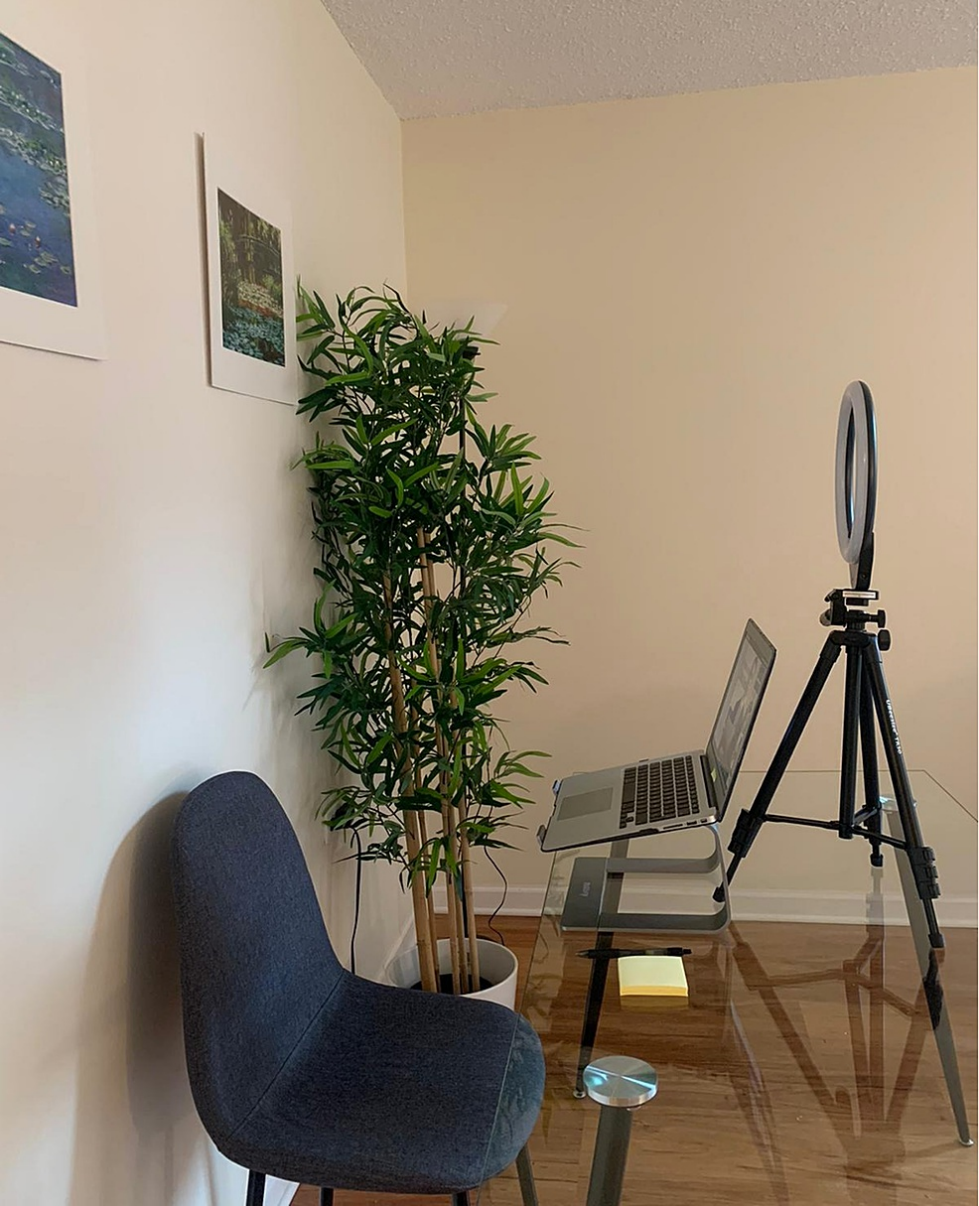
Optimal interview setup The picture depicts an optimal interview setting, which includes a neutral and clean background, proper camera height provided by a laptop stand, and the addition of a ring light to ensure appropriate lighting

Eye contact and optimization of the facial appearance should be ensured by angling the video camera slightly downward before starting the interview. The camera should be placed at eye level to mirror the applicant's view directly into the interviewer's eyes (Figure 1) [6]. The camera resolution should be 720p or higher [7]. Audio is perhaps the most essential element of the interview process. Applicants should consider investing in a webcam that delivers superb audiovisual performance with built-in noise reduction [4]. Using headphones with a microphone can help prevent echoes.

Settings

It is recommended that applicants preferably use any designated interview rooms in libraries, buildings, medical schools, or a quiet, clean, organized, and well-illuminated area with a neutral background at home. Applicants may sit in front of a window, or artificial light devices could be used to ensure proper illumination while avoiding any source of backlighting. Candidates can create a professional background that reveals something about their personalities and demonstrates a highly efficient work environment. It is essential to keep the place free from distractions, including people, pets, phone calls, alarms, doorbells, and pagers [8]. In addition, ergonomic chairs without wheels are better to prevent unintentional or unconscious balancing and swiveling movements that can convey unintended insecurity and unprofessionalism.

Laptop stands or books could help maintain the camera at eye level. It should be ensured that the image transmitted should mirror the one presented in an in-person interview as closely as possible. Head and shoulders should be visible, while 10-20% of the screen space above the head should be kept empty [9]. A clear and neutral background is recommended for the interview. Crowded walls might be highly distractive. Light-colored walls, plants, and even bookshelves could add visual appeal without overwhelming the image frame [9].

Dress code

Candidates should be dressed in the same manner as they would in an in-person interview [8-9]. When it comes to colors, navy blue is highly recommended as this color is associated with qualities such as being "a team player", trustworthy, honest, and credible. Black is a classic color associated with leadership and authority. Gray is a great neutral choice, and it can portray a logical and analytical professional. White shirts are always a good idea, especially when paired with navy, black, or gray slacks [6].

Brown, orange, and red should be avoided as the main apparel colors. While these colors can be appealing as secondary colors in a tie or accessory, they are often associated with unappealing qualities. For example, the color brown has been shown to be associated with boring, simple, and slow, while red can be interpreted as aggressive or rebellious [6].

Additionally, solid colors are highly recommended over patterns. Wearing solid colors helps ensure that your clothes will not be a distraction. However, wearing stripes or busy patterns on videos can create an incredibly distracting distortion and end up creating a strobe-like effect. These wavy lines may appear blurry, making your apparel choice look less professional.

Behavior

Virtual interviews are far more challenging than in-person ones. A fluent interview allows for the assessment of attentiveness, communication, motivation, trustworthiness, and other important factors among participants. Applicants must present a strong, didactic, and participative role in order to capture the attention for future references [10].

Upper body language plays a crucial role in remote interviews. Smiling makes people look confident and comfortable. Hand movements that match strong vocal intonation to emphasize important comments are recommended. To be self-aware of your posture is essential; shoulders should be relaxed and both feet should be on the floor. Nervous fidgeting with pens, glasses, or coins should be avoided. If fidgeting with something cannot be avoided, stress balls that are kept out of frame could be a good solution [10].

It is recommended to speak in a clear and slow manner, with short pauses while answering the questions. This will ensure that interviewers are listening without any inconvenience. Frequently, applicants tend to answer questions very rapidly, triggered by nervousness, leaving blank spaces during the interview, which could feel awkward. To avoid that, answers of suitable length regarding the most common interview questions should be prepared in advance. In addition, having an organized strategy could enable applicants to quickly come up with other answers during the interview [8,10].

## Conclusions

The experience that residency applicants had during the last interview cycle had made us realize how important it is for the applicants to have control over elements related to technology, settings, dress code, and behavior while attending virtual interviews. The awareness that these factors can affect the virtual interview process and optimum preparation by applicants are keys to ensuring a successful interview.
